# Linolenic Acid Inhibits Cancer Stemness and Induces Apoptosis by Regulating Nrf2 Expression in Gastric Cancer Cells

**DOI:** 10.3390/cimb47080646

**Published:** 2025-08-12

**Authors:** Jen-Lung Chen, Yi-Shih Ma, Kuen-Jang Tsai, Hsin-Yi Tsai, Li-Jen Yeh, Hung-Wen Tsai, Judy Yen, Hong-Wen Tsai, Ming-Wei Lin

**Affiliations:** 1 Department of Surgery, E-Da Hospital, I-Shou University, Kaohsiung 82445, Taiwan; sardo0926@gmail.com; 2 Department of Chinese Medicine, E-Da Cancer Hospital, I-Shou University, Kaohsiung 82445, Taiwan; m2367591@ms25.hinet.net; 3 Department of Surgery, E-Da Cancer Hospital, I-Shou University, Kaohsiung 82445, Taiwan; tsai560612@gmail.com; 4 Department of Medical Research, E-Da Cancer Hospital, I-Shou University, Kaohsiung 82445, Taiwan; y7952pipi@gmail.com; 5 Department of Anesthesiology, E-DA Cancer Hospital, I-Shou University, Kaohsiung 82445, Taiwan; ed110880@edah.org.tw; 6 Department of Pathology, National Cheng Kung University Hospital, Tainan 70403, Taiwan; hungwen@mail.ncku.edu.tw; 7 Taipei American School, Taipei 11152, Taiwan; 25judyy@students.tas.tw; 8 Graduate Institute of Patent, National Taiwan University of Science and Technology, Taipei 106335, Taiwan; 9 Department of Nursing, College of Medicine, I-Shou University, Kaohsiung 82445, Taiwan

**Keywords:** linolenic acid, ω-3 polyunsaturated fatty acids, gastric cancer, cancer stemness, Nrf2

## Abstract

Although chemotherapy is the preferred treatment for gastric cancer, the therapeutic drugs currently available have limited efficacy and severe side effects. Cancer stem cells within tumor masses have the distinctive properties of self-renewal, maintenance, and resistance to chemotherapy. Hence, agents capable of targeting stemness in gastric tumors with minimal side effects are urgently required. Enzymes that generate reactive oxygen species contribute to the high oxidation levels observed in tumors. Additionally, nuclear factor erythroid 2-related factor 2 (Nrf2), an antioxidant transcription factor, regulates cancer stemness. Increasing evidence highlights the potential of nutritional supplementation to treat cancer stemness. ω-3 polyunsaturated fatty acids support human health and offer benefits for cancer treatment. Linolenic acid (LA), an ω-3 polyunsaturated fatty acid, inhibits the expression of proteins associated with stemness and promotes apoptosis in gastric cancer cells. Our findings indicated that LA treatment substantially inhibited key characteristics of gastric cancer stemness and induced oxidative stress and caspase-3-mediated apoptosis by downregulating Nrf2-mediated expression. These results suggest that LA is a promising nutritional supplement for targeting cancer stemness in the treatment of gastric cancer.

## 1. Introduction

Gastric cancer is considered one of the deadly types of malignant tumors, with a five-year survival rate of about 20%. In 2020, there were approximately 1.1 million new diagnoses of stomach cancer. Notably, around 75% of these new cases and related deaths occurred in Asia. In 2023, gastric cancer was still the sixth most common cause of cancer-related deaths worldwide [1,2]. Systemic chemotherapy involving multiple drugs is an effective treatment for recurrent gastric cancer. Nevertheless, resistance to chemotherapy in gastric tumors frequently results from the genetic heterogeneity of tumor cells [3]. Additionally, studies have identified cancer stem cells (CSCs) as critical contributors to chemotherapy resistance [4,5,6]. CSCs refer to a subpopulation of cells within tumors that can self-renew, retain stem-like properties, and contribute to cancer recurrence. These attributes render CSCs a promising target for novel anticancer treatments and tailored therapies in precision medicine.

Nuclear factor erythroid 2-related factor 2 (Nrf2) is a crucial transcription factor that maintains cellular oxidative balance. In response to oxidative stress, Nrf2 binds to the promoter regions of antioxidant genes and induces the production of antioxidant enzymes [7]. High Nrf2 expression in CSCs contributes to the cells’ survival and resistance to the oxidative stress induced by chemotherapy and radiotherapy within the tumor microenvironment [8,9]. Nrf2 is also a potential prognostic indicator in patients with gastric adenocarcinoma [10,11], rendering it a promising target for cancer therapies. Cancer cells generally have higher levels of reactive oxygen species (ROSs) than normal cells do. Because they have high antioxidant capacity, CSCs keep their ROS levels low, preserving their stem-like properties and enhancing their survival and drug resistance [7]. Studies have identified the surface adhesion molecule CD44 as a marker of gastric CSCs implicated in cancer progression [12,13]. Activation of CD44 or Nrf2 regulates stem cell traits in several cancers, including gastric cancer. Under chemotherapy, Nrf2 may also influence apoptosis in CD44^+^ CSCs [14,15].

Increasing recognition of the health benefits associated with nutritional supplementation has prompted research into the mechanisms underlying these benefits. For example, studies have demonstrated that supplementation with ω-3 polyunsaturated fatty acids derived from fish oil or included in immunonutrition formulations can reduce inflammatory markers or contribute to the recovery of immune function in patients with gastric cancer who are undergoing surgery [16,17,18]. Consequently, a critical evaluation of nutritional supplements that exert therapeutic effects during chemotherapy for gastric cancer and do not have adverse side effects is urgently required. Linoleic acid (LA), an ω-3 polyunsaturated fatty acid, was reported to suppress inflammation and oxidative stress [19,20]. However, the mechanisms underlying the anticancer effects of LA through inhibition of gastric cancer stemness are unclear. The present study assessed the effects of LA on stemness in gastric cancer cells and hypothesized that LA suppresses gastric cancer stemness by downregulating Nrf2.

## 2. Materials and Methods

### 2.1. Cell Culture and Reagent

The human gastric cancer cell line, MKN45, was purchased from DSMZ (ACC-409, DSMZ, Braunschweig, Germany) and cultured using RPMI1640 (Gibco, Waltham, MA, USA) medium with 10% fetal bovine serum (Gibco, Waltham, MA, USA), 1% penicillin/streptomycin (Gibco, Waltham, MA, USA) under 5% CO_2_ at 37 °C. The LA (ω-3; TargetMol, Washington Street, Wellesley Hills, MA, USA), brusatol (Nrf2 inhibitor, Sigma-Aldrich, St. Louis, MO, USA), AI-1 (Focus biomolecules, Plymouth Meeting, Davis Drive, PA, USA), and LY294002 (Akt inhibitor, Abcam, Cambridge, UK) in dimethyl sulfoxide (DMSO) (Sigma-Aldrich, St. Louis, MO, USA) were prepared and dissolved in culture medium before treatment.

### 2.2. Flow Cytometry Analysis

After treatment, the cells were washed with cold phosphate-buffered saline (PBS) and stained with surface marker antibody. After staining, cells were washed twice by cold PBS before analysis. The CSC marker (CD44; BD Biosciences, San Jose, CA, USA) or isotype control antibody (IgG1, κ Isotype Control; BD Biosciences, San Jose, CA, USA) expression on the human gastric cancer cells was analyzed through flow cytometry. The isotype control antibody serves as a negative control to help distinguish between specific and non-specific antibody binding. The cells stained with the isotype control antibody are regarded as background staining.

For mitochondrial oxidative stress analysis, the MKN45 cells were stained using MitoSOX™ Mitochondrial Superoxide Indicators (Invitrogen, Waltham, MA, USA) and analyzed through flow cytometry. The unstained cells serve as a background reference.

For ROS generation analysis, the MKN45 cells were stained using Dihydroethidium (Invitrogen, Waltham, MA, USA) and analyzed through flow cytometry. The unstained cells serve as a background reference.

For caspase activity analysis, the human gastric cancer cells were stained with the Cleaved Caspase-3 Staining Kit (Abcam, Cambridge, UK) by using FITC-DEVD-FMK. FITC- DEVD-FMK is a non-toxic, cell-permeable fluorescent substrate that irreversibly binds to activated caspase-3 in apoptotic cells. Detection of the labeled cells was determined by flow cytometry.

### 2.3. Nuclear Protein Isolation

The MKN45 cells were seeded in a 100 mm Petri dish at 1 × 10^6^ cells/dish and cultured for 24 h before treatment. Cells were harvested, and nuclear fractions were isolated, using a commercially available NE-PER™ Nuclear and Cytoplasmic Extraction Kit (Thermo Fisher Scientific, Waltham, MA, USA) according to the instructions provided by the manufacturer. The lamin B1 was used as loading control for the nuclear protein.

### 2.4. Western Blot Analysis

The MKN45 cells were washed with PBS. Total proteins were extracted, and protein concentrations were measured using the Bio-Rad Bradford Protein Assay (Bio-Rad, Hercules, CA, USA). Equal quantities of total proteins were separated through BOLT BISTRIS PLUS 4–12% sodium dodecyl sulfate–polyacrylamide gel electrophoresis (Thermo Scientific, New York, NY, USA) and transferred onto polyvinylidene fluoride membranes. The membranes were blocked with a blocking buffer (Bio-Rad, Hercules, CA, USA) for 30 min at room temperature and incubated with the primary antibodies: CD44 (1:1000; ABclonal, Woburn, MA, USA), Nrf2 (1:1000; ABclonal, Woburn, MA, USA), SOX2 (1:1000; ABclonal, Woburn, MA, USA), HO-1 (abcam, Cambridge, UK), Akt (1:1000; Cell Signaling, Danvers, MA, USA), phosphor-Akt (1:1000; Cell Signaling, Danvers, MA, USA), LaminB1 (1:1000; Cell Signaling, Danvers, MA, USA), β-actin (1:5000; Cell Signaling, Danvers, MA, USA) at 4 °C after the membranes were washed with PBS with Tween 20. The membranes were incubated with secondary antibodies at room temperature for 1 h and then analyzed using an enhanced chemiluminescence detection system.

### 2.5. Statistical Analysis

All data were analyzed by GraphPad Prism (version 8). All graphs in figures were presented as means ± standard error of measurement (SEM). Statistical analysis was performed using Student’s *t*-test or one-way ANOVA analysis to compare data between two groups. All analyses with statistical significance were set at *p* < 0.05. Statistical results were labeled in each figure as follows: * *p* < 0.05, ** *p* < 0.01, *** *p* < 0.001.

## 3. Results

### 3.1. LA Inhibits Cancer Stemness Marker CD44 in Gastric Cancer Cells

**Figure 1:**
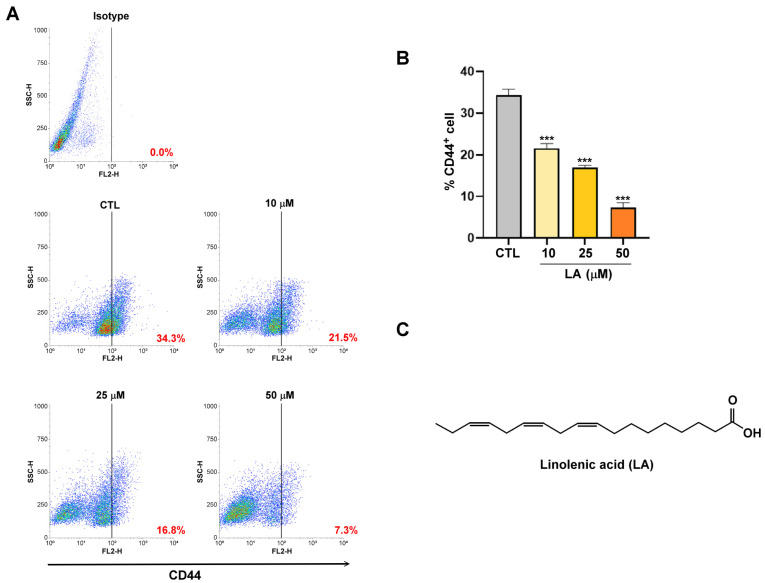
(**A**) MKN45 cells were treated with linoleic acid (LA; 10, 25, or 50 μM) for 72 h. CD44^+^ cells were analyzed through flow cytometry. MKN45 cells were also stained with an isotype control antibody to serve as a background reference. (**B**) Quantification of CD44^+^ cells after LA (10, 25, or 50 μM) treatment. (**C**) Structural formula of LA. Data are presented as the mean ± standard error of the mean; *n* ≥ 3 independent experiments; two-tailed Student’s *t* test. *** *p* < 0.005.

CD44 expression is correlated with redox balance and chemoresistance and is a promising target for enhancing the prognosis and treatment of various cancers [21,22]. As shown in Figure 1A,B, the population of CD44^+^ cancer stem cells in the control (CTL) human gastric cancer MKN45 cells is 34.3%. However, when these cells were treated with varying concentrations of LA (10, 25, and 50 μM), the proportion of CD44^+^ cancer stem cells decreased significantly, dropping from 21.5% to 7.3% as observed in flow cytometry analysis. This suggests that LA effectively inhibits the stemness of gastric cancer. The chemical structure of LA is shown in Figure 1C.

### 3.2. LA Inhibits Protein Expression Linked to Gastric Cancer Stemness Through the Nrf2-Mediated Pathway

**Figure 2:**
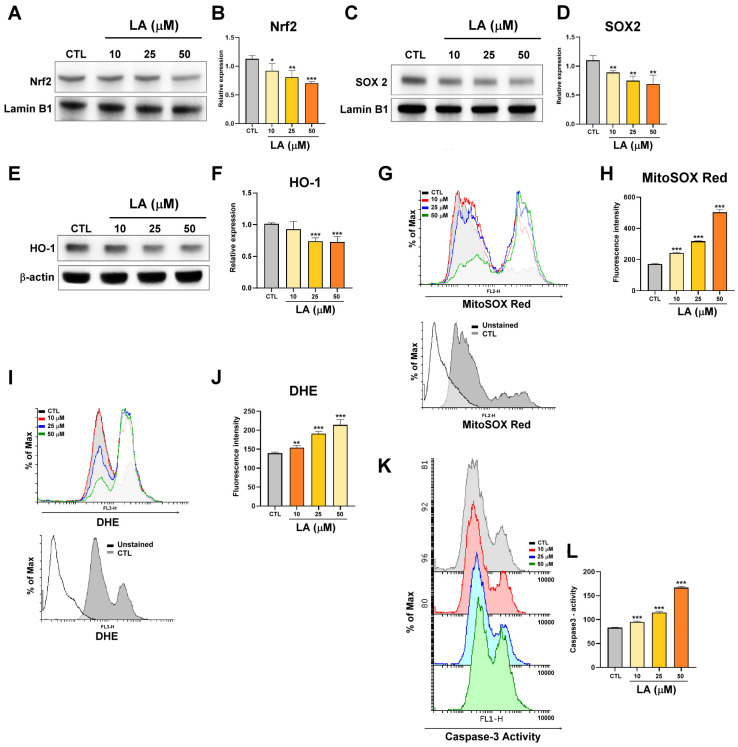
(**A**) Nrf2 expression and (**B**) quantification of Nrf2 expression in nuclear fractions isolated from MKN45 cells, analyzed through Western blotting, after their treatment with LA (10, 25, or 50 μM) for 72 h. Lamin B1 served as the loading control. (**C**) SOX2 expression and (**D**) quantification of SOX2 expression in the nuclear fractions of MKN45 cells, analyzed through Western blotting, after their treatment with LA (10, 25, or 50 μM) for 72 h. Lamin B1 served as the loading control. (**E**) HO-1 expression and (**F**) quantification of HO-1 expression in MKN45 cells after their treatment with LA (10, 25, or 50 μM) for 72 h. (**G**,**H**) Mitochondrial oxidative stress in MKN45 cells, analyzed through mitoSOX red staining and flow cytometry, after their treatment with vehicle (CTL: black line) or LA (10, 25, or 50 μM; 10 μM: red line; 25 μM: blue line; 50 μM: green line)) for 72 h. The unstained cells (Unstained) serve as a background reference. The cells that were stained with mitoSOX red and treated with the vehicle act as the control group (CTL). (**I**,**J**) Reactive oxygen species generation in MKN45 cells, analyzed through dihydroethidium staining and flow cytometry, after their treatment with vehicle (CTL: black line) or LA (10, 25, or 50 μM; 10 μM: red line; 25 μM: blue line; 50 μM: green line) for 72 h. The unstained cells (Unstained) serve as a background reference. The cells that were stained with DHE and treated with the vehicle act as the control group (CTL). (**K**,**L**) Caspase-3 activity in MKN45 cells, analyzed through flow cytometry, after their treatment with LA (10, 25, or 50 μM) for 72 h. Data are presented as the mean ± standard error of the mean; *n* ≥ 3 independent experiments; two-tailed Student’s *t* test. * *p* < 0.05, ** *p* < 0.01, *** *p* < 0.005.

Nrf2 regulates the expression of antioxidative genes. SOX2 (SRY-related high mobility group box 2) is a transcription factor involved in cancer stemness [23]. To determine whether LA suppresses gastric cancer stemness by modulating Nrf2 or SOX2 activation, this study isolated nuclear proteins from MKN45 cells after they were treated with LA. The results demonstrated that LA (10, 25, or 50 μM) inhibited Nrf2 and SOX2 nuclear translocation in a dose-dependent manner (Figure 2A–D). Heme oxygenase-1 (HO-1), a downstream antioxidant protein regulated by Nrf2, was also suppressed by LA (Figure 2E,F). Additionally, LA induced mitochondrial oxidative stress (Figure 2G,H), increased intracellular ROS levels (Figure 2I,J), and activated caspase-3-mediated apoptosis (Figure 2K,L) in a dose-dependent manner.

**Figure 3:**
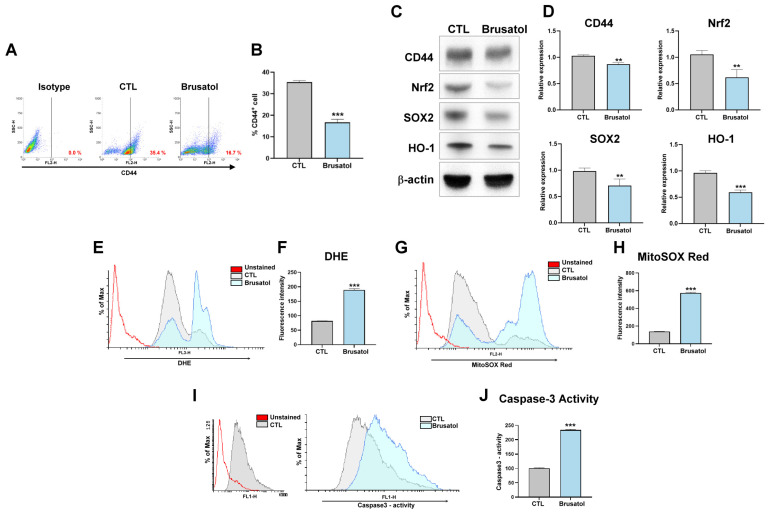
(**A**,**B**) CD44^+^ cell population in MKN45 cells, analyzed through flow cytometry, after their treatment with brusatol (40 nM) for 72 h. MKN45 cells were also stained with an isotype control antibody to serve as a background reference. (**C**) CD44, Nrf2, SOX2, HO-1, and β-actin expression and (**D**) quantification of the expression of these proteins in MKN45 cells after their treatment with brusatol (40 nM) for 72 h. (**E**,**F**) ROS generation in MKN45 cells, analyzed through dihydroethidium staining and flow cytometry, after their treatment with brusatol (40 nM) for 72 h. (**G**,**H**) Mitochondrial oxidative stress in MKN45 cells, analyzed through mitoSOX red staining and flow cytometry, after their treatment with brusatol (40 nM) for 72 h. (**I**,**J**) Caspase-3 activity in MKN45 cells, analyzed through flow cytometry, after their treatment with brusatol (40 nM) for 72 h. Data are presented as the mean ± standard error of the mean; *n* ≥ 3 independent experiments; two-tailed Student’s *t* test ** *p* < 0.01, *** *p* < 0.005.

To investigate the role of Nrf2 in gastric cancer stemness, the Nrf2 inhibitor brusatol was employed to assess stemness and the expression of antioxidative proteins. As illustrated in Figure 3A,B, brusatol treatment (40 nM) substantially reduced the size of the CD44^+^ gastric cancer stem cell population. The proportion of CD44^+^ cancer stem cells decreased significantly, dropping from 35.4% to 16.7%. Specifically, brusatol inhibited the expression of CD44, Nrf2, SOX2, and HO-1 (Figure 3C,D). Additionally, brusatol increased the intracellular ROS levels and mitochondrial oxidative stress observed through flow cytometry (Figure 3E–H). Inhibition of Nrf2 by brusatol also induced apoptosis in gastric cancer cells through caspase-3 activation (Figure 3I,J).

**Figure 4:**
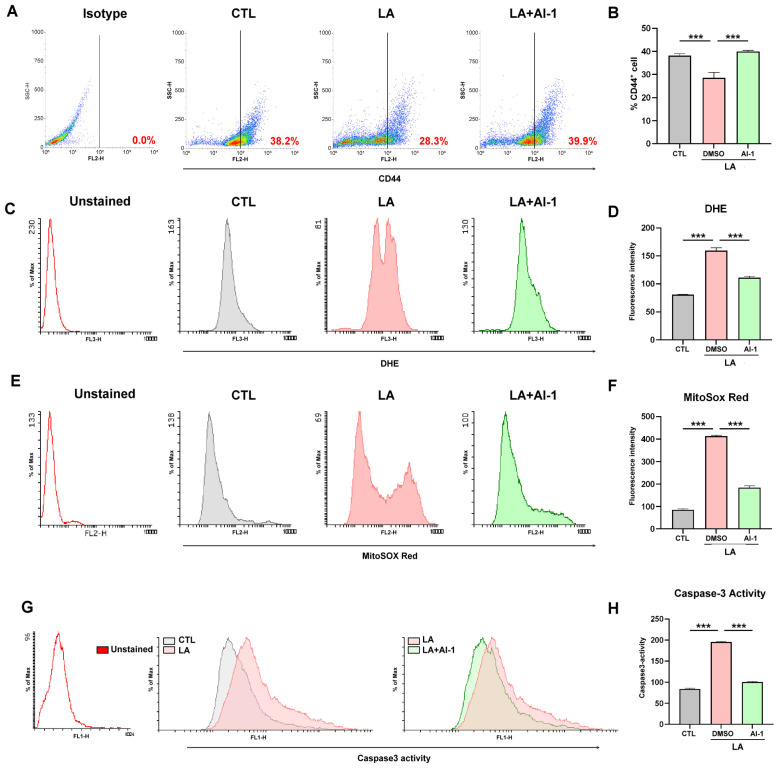
(**A**,**B**) CD44^+^ cell population, analyzed through flow cytometry, in MKN45 cells treated with an LA (25 μM) vehicle, or an LA (25 μM) vehicle + AI-1 (10 μM) for 72 h. MKN45 cells were also stained with an isotype control antibody to serve as a background reference. (**C**,**D**) ROS generation, (**E**,**F**) mitochondrial oxidative stress, and (**G**,**H**) caspase-3 activation, observed through flow cytometry analysis, in MKN45 cells treated with an LA (25 μM) vehicle or an LA (25 μM) vehicle + Nrf2 activator AI-1 (10 μM) for 72 h. Data are presented as the mean ± standard error of the mean; *n* ≥ 3 independent experiments; two-tailed Student’s *t* test. *** *p* <0.005.

To confirm that LA suppresses stemness and induces oxidative-stress-mediated apoptosis in MKN45 gastric cancer cells through Nrf2-dependent mechanisms, the present study used the Nrf2 activator AI-1 to evaluate CD44 expression, oxidative stress, and caspase-3 activation under LA treatment. As indicated in Figure 4A–H, AI-1 reversed LA-induced CD44 suppression, ROS increases, mitochondrial oxidative stress, and caspase-3 activation.

### 3.3. LA Regulates Nrf2 Expression Through the Akt Signaling Pathway

**Figure 5:**
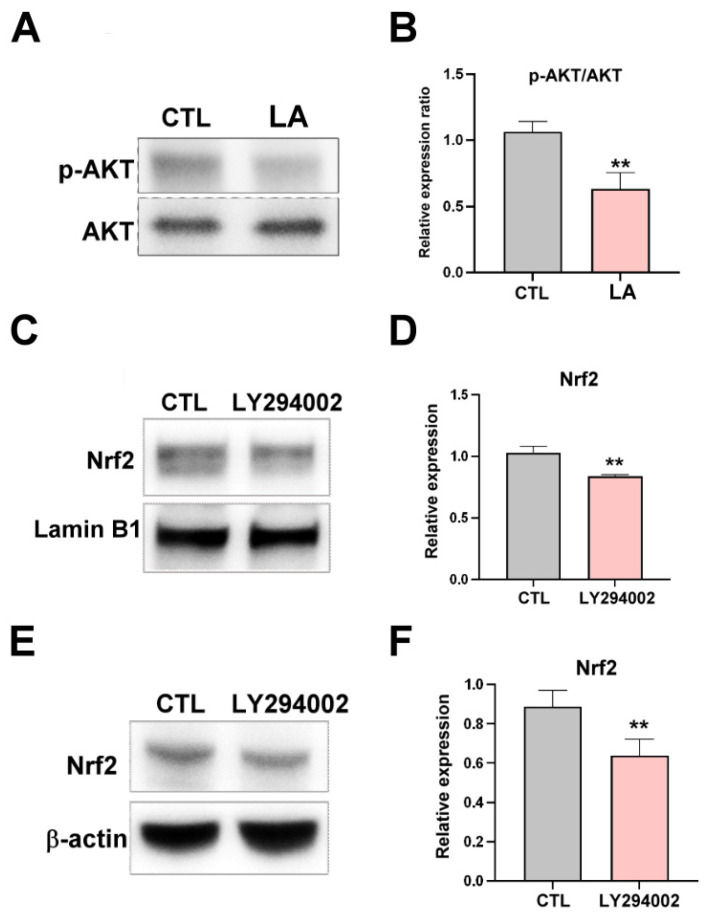
(**A**) Expression of phosphorylated-Akt and total Akt in MKN45 cells, analyzed through Western blotting, after their treatment with LA (25 μM) for 72 h. (**B**) Quantification of the phosphorylated-Akt/Akt ratio after cells’ treatment with LA (25 μM). (**C**,**D**) Nrf2 and Lamin B1 expression in nuclear fractions isolated from MKN45 cells and analyzed through Western blotting after the cells’ treatment with Akt inhibitor LY294002 (10 μM) for 1 h. (**E**) Nrf2 expression and (**F**) quantification of Nrf2 expression in MKN45 cells after their treatment with LY294002 (10 μM) for 72 h. Data are presented as the mean ± standard error of the mean; *n* ≥ 3 independent experiments; two-tailed Student’s *t* test. ** *p* < 0.01.

Akt signaling is involved in gastric cancer stemness characteristics, and activation of the Akt signaling pathway has been reported to regulate the Nrf2-mediated pathway [24]. To determine whether Akt signaling participates in LA-mediated Nrf2 inactivation, the present study employed the Akt inhibitor LY294002. As illustrated in Figure 5A,B, LA inhibited Akt phosphorylation. Specifically, the inhibition of Akt signaling by LY294002 suppressed Nrf2 nuclear translocation (Figure 5C,D) and downregulated Nrf2 expression (Figure 5E,F) in MKN45 human gastric cancer cells. These results suggest that LA suppresses Nrf2 activation by inhibiting Akt phosphorylation.

## 4. Discussion

Cancer stemness refers to the unique ability of certain cancer cells to self-renew and resist chemotherapy. The cell-surface adhesion molecule CD44 has been identified as a marker of gastric cancer stemness and may play a role in cancer development, particularly when exacerbated by *Helicobater pylori* infection [25,26]. Chen et al. first demonstrated the presence of CD44^+^ cells in human gastric tumors and their chemoresistance and stemness [27]. CD44 expression is linked to poorer clinical outcomes in patients with gastric cancer [28,29,30,31]. In a gastric tumor xenograft animal model, CD44^+^ cells exhibited cancer stem cell characteristics, and CD44 suppression led to decreased tumor growth [32]. Consequently, targeting cancer stemness could be a promising strategy for preventing tumor development or delaying tumor progression [33].

Nrf2 is a crucial transcription factor that regulates antioxidant response and maintains cellular redox hemostasis. Under oxidative stress, Nrf2 translocates to the nucleus and activates genes related to antioxidants. Studies have indicated that Nrf2 may contribute to cancer stemness [7,8]. Gastric cancer cells promote stemness traits through Akt/Nrf2 signaling pathways [14,34]. SOX2 is an additional transcription factor associated with stemness. Moreover, notum is a carboxylesterase highly expressed in early-stage gastric cancer that has been reported to enhance gastric cancer stemness through Akt/SOX2 signaling [35]. However, the role of antioxidant transcription factors in regulating gastric cancer stemness remains unclear. One study demonstrated that luteolin-mediated downregulation of Nrf2 suppressed stemness in triple-negative breast cancer cells [36]. The present study revealed that Nrf2 activation regulates SOX2 expression in gastric cancer cells. Similarly, Srinivasan et al. indicated that CD44 regulates SOX2 expression in prostate cancer cells and proposed SOX2 as a downstream target of CD44 [37]. HO-1 is a downstream antioxidant protein of Nrf2 that contributes to tumors’ growth, progression, and therapy resistance. High HO-1 expression is linked to stem-cell-like properties in various cancers, and HO-1 inhibition reduces expression of CD44, a stemness marker [38,39]. The present study evaluated the levels of proteins associated with stemness after LA treatment of gastric cancer cells and revealed that LA suppresses cancer stemness through the Akt/Nrf2/CD44/SOX2 signaling axis. These findings underscore the therapeutic potential of nutritional supplements to enhance gastric cancer care.

Studies have increasingly recognized the potential of nutritional supplementation to support overall human health and enhance cancer care. Specifically, research suggests that ω-3 polyunsaturated fatty acids such as LA may reduce cancer risk by modulating genetic pathways related to inflammation, oxidative stress, and tumor cell apoptosis [40]. One such polyunsaturated fatty acid, docosahexaenoic acid, affects mitochondrial function and induces ROS overproduction in prostate cancer cells [41]. The other ω-3, docosahexaenoic acid (DHA), also induces substantial oxidative stress in mitochondria and accelerates adenosine triphosphate depletion by inhibiting superoxide dismutase in cancer cells [42]. In the present study, LA selectively induced oxidative stress in cancerous mitochondria and promoted ROS overproduction in gastric cancer cells by inhibiting the anti-oxidative transcription factor Nrf2. This inhibition was associated with reduced expression of proteins linked to stemness and increased caspase-3-mediated apoptosis. LA has been reported to inhibit proliferation of gastric cancer cells [40] and regulate the growth of cervical cancer [43]. Similarly to LA, DHA, was also shown to reduce the viability of gastric cancer cells, specifically MKN45, and to improve their sensitivity to chemotherapeutic agents. Comparable results were observed in another gastric cancer cell line, AGS (Supplementary Figure S1–S3). Higher dietary intake of LA is also associated with a lower risk of colorectal cancer [44]. Nutraceuticals offer a novel approach to cancer care, providing potential benefits in inhibiting carcinogenesis. However, unlike conventional anticancer agents, LA exerts anti-inflammatory and antioxidant effects in normal cells and exerts protective effects in animal models of gastric injury by reducing levels of ROSs [45]. Interestingly, previous study showed that LA and DHA effectively reduce oxidative stress via the activation of the Nrf2 pathway [46]. Taking ω-3 polyunsaturated fatty acids shows great potential as a new strategy for preventing cancer [47,48]. A possible way that a diet rich in ω-3 polyunsaturated fatty acids could enhance the tumor-suppressing effects of chemotherapeutic agents is by regulating oxidative stress levels.

## 5. Conclusions

The findings of the present study suggest that LA considerably suppresses key features of gastric cancer stemness through the Akt/Nrf2/CD44/SOX2 signaling pathways. Therefore, LA has therapeutic potential as a nutritional supplement to target stemness in the treatment of gastric cancer.

## Data Availability

All data sets generated or analyzed in this study were included in the published article. Detailed data sets supporting the current study are available from the corresponding author upon request. This study did not generate new codes.

## Supplementary Materials

The following supporting information can be downloaded at: https://www.mdpi.com/article/10.3390/cimb47080646/s1.

## Abbreviations

The following abbreviations are used in this manuscript:

CSC	Cancer stem cell
HO-1	heme oxygenase-1
LA	linolenic acid
Nrf2	nuclear factor erythroid 2-related factor 2
SOX2	SRY-box transcription factor 2
