# Supplementary material for: Linolenic Acid Inhibits Cancer Stemness and Induces Apoptosis by Regulating Nrf2 Expression in Gastric Cancer Cells
Source: Curr Issues Mol Biol. 2025 Aug 12;47(8):646. doi: 10.3390/cimb47080646 (PMC12384521; doi:10.3390/cimb47080646)
Supplement: Supplementary file 1 [file cimb-47-00646-s001.zip › cimb-3754821-supplementary.pdf]

Supplementary Figure

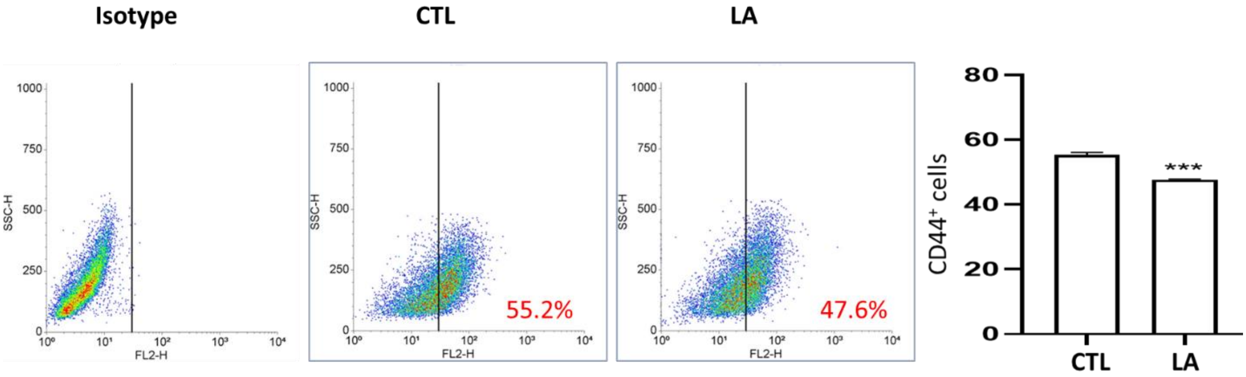

Figure S1. LA inhibits CD44⁺ cancer stem cell populations in human gastric cancer AGS cells. AGS cells were treated with linoleic acid (LA; 10 μM) for 72 h. CD44⁺ cells were analyzed through flow cytometry. AGS cells were also stained with an isotype control antibody to serve as a background reference. (B) Quantification of CD44⁺ cells after LA (10 μM) treatment. Data are presented as the mean ± standard error of the mean; *n* ≥ 3 independent experiments; two-tailed Student's *t* test. *** *p* < 0.005.

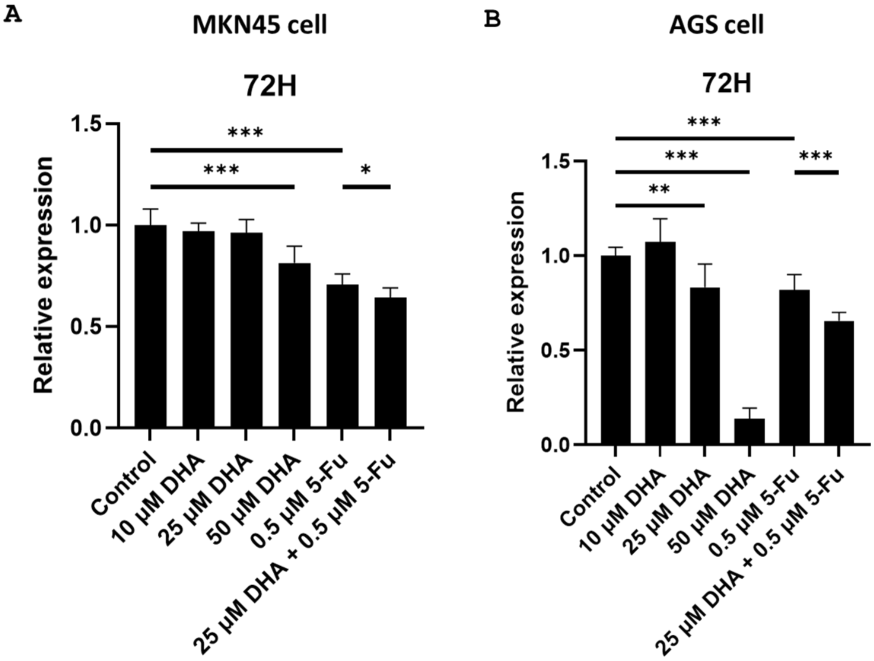

Figure S2. DHA inhibits cell viability and enhances chemosensitvity in human gastric cancer, MKN45 and AGS cells. MKN45 or AGS cells were treated with DHA (10, 25, 50 μM) or chemotherapeutic agent, 5-FU (0.5 μM), for 72 h. Cell viability were evaluated by Cell Counting Kit-8. Data are presented as the mean ± standard error of the mean;

$n \geq 3$ independent experiments; two-tailed Student's t test. * $p < 0.05$, ** $p < 0.01$, *** $p < 0.005$.

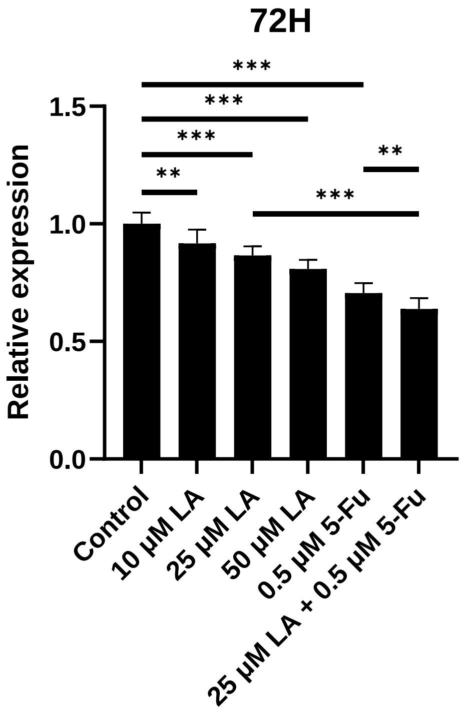

Figure S3. LA inhibits cell viability and enhances chemosensitvity in human gastric cancer, MKN45 cells. MKN45 cells were treated with LA (10, 25, 50 μM) or chemotherapeutic agent, 5-FU (0.5 μM), for 72 h. Cell viability were evaluated by Cell Counting Kit-8. Data are presented as the mean ± standard error of the mean; $n \geq 3$ independent experiments; two-tailed Student's t test. ** $p < 0.01$, *** $p < 0.005$.

Cell viability analysis

Human gastric cancer cells were seeded in 96-well dishes in quadruplicate at 6000 cells/well and cultured for 24 h before treatment. Cell viability was analyzed using Cell Counting Kit-8 (Sigma–Aldrich, St. Louis, MO, USA), and absorbance was measured at 450 nm by using a microplate reader.
